# A low-cost system for monitoring pH, dissolved oxygen and algal density in continuous culture of microalgae

**DOI:** 10.1016/j.ohx.2022.e00353

**Published:** 2022-08-27

**Authors:** Dung Kim Nguyen, Huy Quang Nguyen, Huyen Thuy T. Dang, Viet Quoc Nguyen, Linh Nguyen

**Affiliations:** a Faculty of Engineering, Vietnam National University of Agriculture, Hanoi 10000, Viet Nam; b Institute of Innovation, Science and Sustainability, Federation University, Churchill, VIC 3842, Australia

**Keywords:** Microalgae, Continuous culture system, Microalgal density, pH, Dissolved oxygen

## Abstract

In a continuous and closed system of culturing microalgae, constantly monitoring and controlling pH, dissolved oxygen (DO) and microalgal density in the cultivation environment are paramount, which ultimately influence on the growth rate and quality of the microalgae products. Apart from the pH and DO parameters, the density of microalgae can be used to contemplate what light condition in the culture chamber is or when nutrients should be supplemented, which both also decide productivity of the cultivation. Moreover, the microalgal density is considered as an indicator indicating when the microalgae can be harvested. Therefore, this work proposes a low-cost monitoring equipment that can be employed to observe pH, DO and microalgal density over time in a culture environment. The measurements obtained by the proposed monitoring device can be utilized for not only real-time observations but also controlling other sub-systems in a continuous culture model including stirring, ventilating, nutrient supplying and harvesting, which leads to more efficiency in the microalgal production. More importantly, it is proposed to utilize the off-the-shelf materials to fabricate the equipment with a total cost of about 513 EUR, which makes it practical as well as widespread. The proposed monitoring apparatus was validated in a real-world closed system of cultivating a microalgae strain of *Chlorella vulgaris*. The obtained results indicate that the measurement accuracies are 0.3%, 3.8% and 8.6% for pH, DO and microalgae density quantities, respectively.

## Graphical abstract

**Figure:**
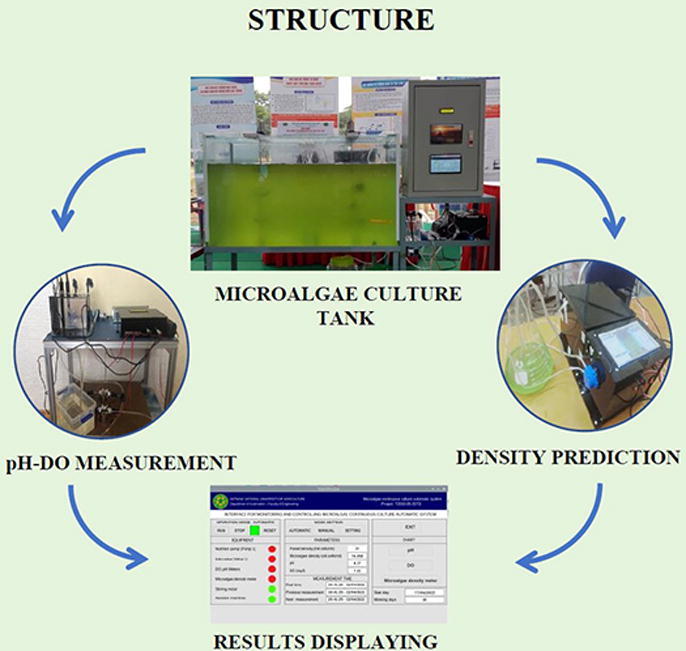

**Specifications table**

**Table:** 

Hardware name	A monitoring system for continuous culture of microalgae
Subject area	• Engineering and material science
Hardware type	• Imaging tools
	• Field measurements and sensors
	• Electrical engineering and computer science
Closest commercial analog	No commercial analog is available.
Open source license	CC-By Attribution 4.0 International
Cost of hardware	513.08 EUR
Source file repository	https://doi.org/10.17605/OSF.IO/HDYCE

## 1 Hardware in context

Microalgae play a particularly important role in the food chain of the aquatic environment. In the early stages, the aquatic larvae are often very small, extremely fragile and not fully physiologically developed while their cognitive organs and digestive systems are immature. These cause limitations in the selection and use of appropriate food. Studies have shown that fresh microalgae are a particularly important food for aquatic larvae [1] because of their properties consistent with the biological and physiological characteristics of the larvae. The survival rate of larvae fed with fresh algae is much higher than that of others fed with commercial feeds [1]. Microalgae are also used to produce zooplankton, a type of food for larval and early juvenile stages of crustaceans and fish. Therefore, it is necessary to cultivate microalgae in aquaculture hatcheries [2].

In fact, algae can be produced by using various methods including batch, semi-continuous and continuous models as presented in the context of the phytoplankton culture [1]. In the batch culture, all algae are cultivated and harvested at once when their density reaches maximum. Though the method is simple and flexible, it is difficult to predict quality of the harvested products since they are not continuously monitored during the growth. Moreover, it is quite costly as significant labor is needed in terms of manually cleaning, sterilizing, refilling, inoculating and harvesting. In contrast to the batch culture, in the semi-continuous cultivation, microalgae are partially harvested in periods. After each harvesting, more seawater and nutrient are added to the cultivating environment for stable growth of algae over time. However, the species are easily contaminated by competitors, predators or metabolites. So far, the best model of cultivating microalgae is the continuous culture where the fertilized seawater is continuously supplied to a culturing chamber in a closed cultivation system such as photobioreactors. Furthermore, in the continuous cultivation system, some parameters in the culturing environment including microalgal density, pH or dissolved oxygen (DO) can be monitored and controlled over time, which ultimately maintains growth of algae at maximum rate. In other words, being able to monitor and control those environmental parameters leads to quality of the microalgae productions predictable and reliable. In parallel, biomass growth of algae can also be observed [3].

Due to its advantages, the continuous culture system has attracted particular interest in the microalgae cultivation community, from both researchers and practitioners [4], [5], [6]. For instance, Laing in [7] proposed a 40 liter continuous system that aims to maintain biomass concentration in culturing flagellates. Another continuous cultivation system was designed and built by Sananurak et al. in [8] to produce two types of microalgae, *Tetraselmis suecica and Brachionus plicatilis*, for larval fish. By exploiting solid-state materials, Naumann et al. developed a twin-layer continuous photobioreactor [9] for cultivating four strains of microalgae including *Isochrysis sp. T.ISO, Tetraselmis suecica, Phaeodactylum tricornutum, Nannochloropsis sp.*, used as live feeds in hatcheries. It is noted that the premise behind efficiency of the continuous culture system in producing microalgae is that the environmental parameters in the culturing environment are required to be continuously monitored and controlled [10]. For instance, the author of the work [11] proposed an optimized up-scaled photobioreactor for continuously cultivating algal strain *S. platensis*, where critical parameters including liquid level, pH condition and temperature during the culture operations are remotely monitored through smartphone application. A recent review paper [12] provides discussions about exploiting advanced technologies such as Internet of things (IoT) or artificial intelligence in smartly enhancing productivity and product quality of microalgae farming. Apparently, IoT can be a good platform for continuously monitoring the cultivation parameters in remote operation modes [13], particularly paramount for smart manufacturing setup [14].

Apart from the nutrients being constantly supplemented, other dominant environmental parameters in the cultivating medium deciding growth and quality of microalgae and being required to be strictly controlled are pH, DO and density of algae. In order to efficiently control those environmental parameters, automatically monitoring them over time is highly expected since manually taking samples from the culture chamber and analysing those information in a laboratory is particularly impractical for a closed and continuous cultivation system. More importantly, continuous feedback from a monitoring mechanism is necessary for an autonomous control system. Therefore, this work proposes a low-cost equipment that can be employed to constantly monitor pH, DO and microalgal density in a continuous culture system. It is noted that density of microalgae directly influences on light conditions in the culturing environment. Together with pH and DO, light condition is needed in photosynthesis of microalgae and ultimately drives growth rate, biomass concentration and quality of the microalgae products [15], [16]. On the other hand, in the continuous culture mode, information of microalgal density is also utilized to either control the nutrient supplement processes or decide when microalgae can be harvested. Therefore, it is critical to constantly monitor pH, DO and microalgal density over time in a closed cultivation system.

## 2 Hardware description

**Fig. 1:**
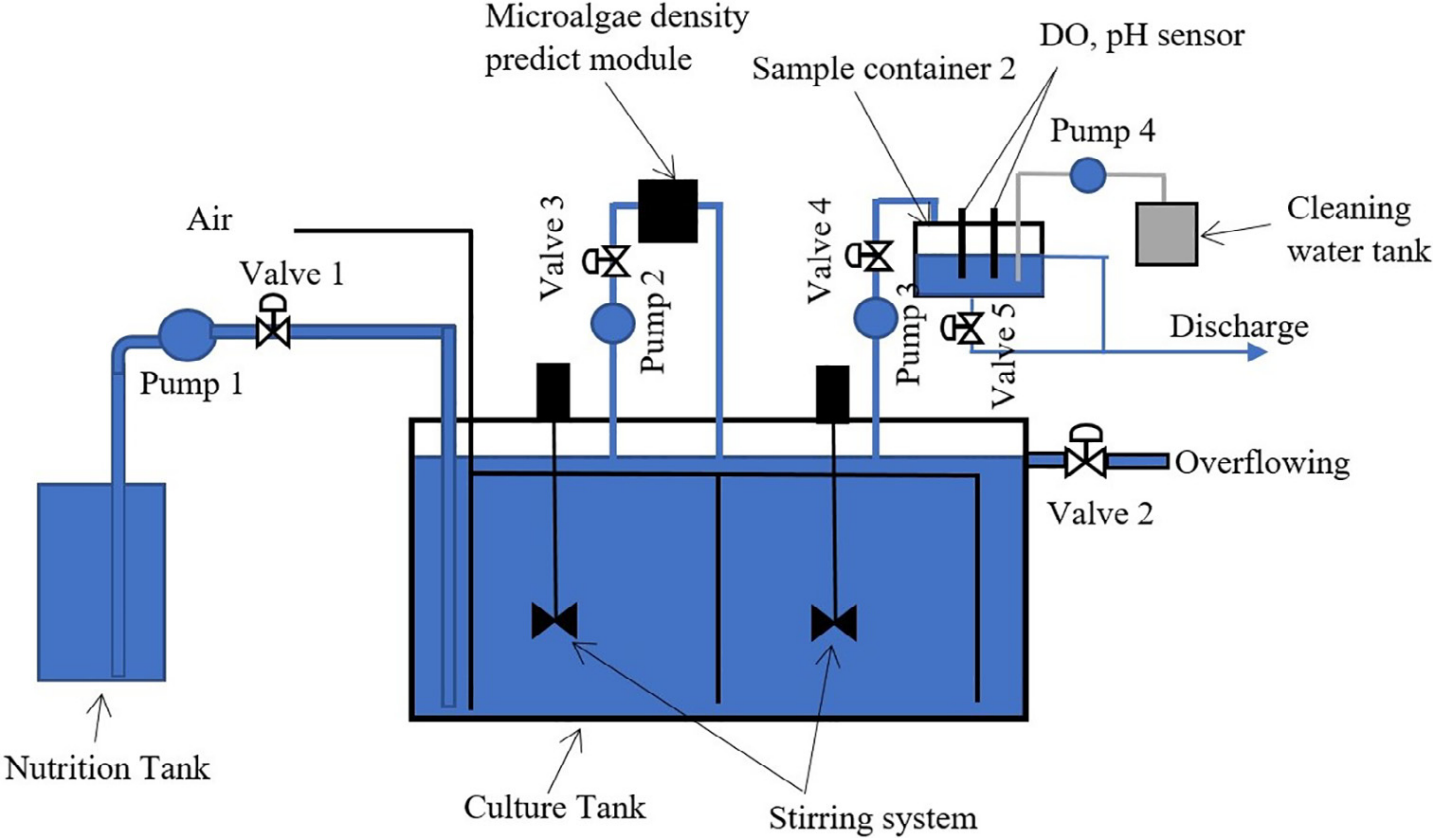
A model of the continuous system for culturing microalgae.

Before describing components of the proposed monitoring devices for constantly observing pH, DO and microalgal density in the cultivation environment, it briefly introduces a model of a continuous system for culturing microalgae as schematically demonstrated in Fig. 1. As can be seen that the system includes a culture tank, which is also known as a growth chamber. The culture tank is sequentially supplemented the nutrients through the fresh medium from the nutrition tank by Pump 1 whenever density of the microalgae in the growth chamber reaches to a preset threshold. Since microalgae absorb the nutrition over time for their continuous growth, the nutrients in the cultivation tank gradually reduce and the supplement is necessary in a continuous mode. It is noted that when Pump 1 is operating, Valve 2 is also open, which allows some microalgae to be harvested through the overflowing solution.

In addition to the nutrient supplement, the culture tank is equipped a ventilation system that not only provides air for the photosynthesis of microalgae but also supplies oxygen to the growth solution. Moreover, a stirring mechanism is additionally deployed to generate a continuous flow of the solution in the culture chamber, preventing microalgae from being deposited on the tank bottom. Hardware of the proposed monitoring system is now delineated as follows.

### 2.1 Microalgal density monitoring

**Fig. 2:**
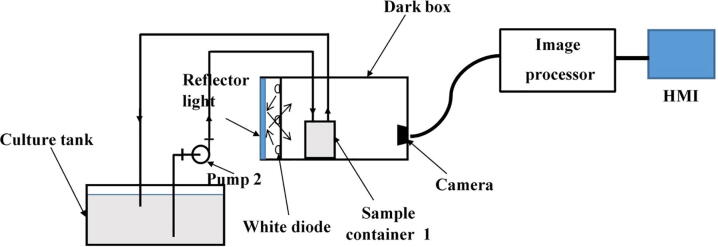
A schematic diagram of the *microalgae density predict module*.

In order to observe density of microalgae in the culture tank, a *microalgae density predict module* was designed as can be seen in Fig. 1. The microalgal solution is pumped to the module by Pump 2 from the culture tank through Valve 3. In this design, a Kamoer peristaltic pump was chosen whose flow rate ranges from 5.2 ml to 90 ml per minute and a PURO-XD-12VDC solenoid valve. A schematic diagram of the *microalgae density predict module* is illustrated in Fig. 2. As can be seen that the module comprises three main components, a dark box, an image processor and a human machine interface (HMI).

**Fig. 3:**
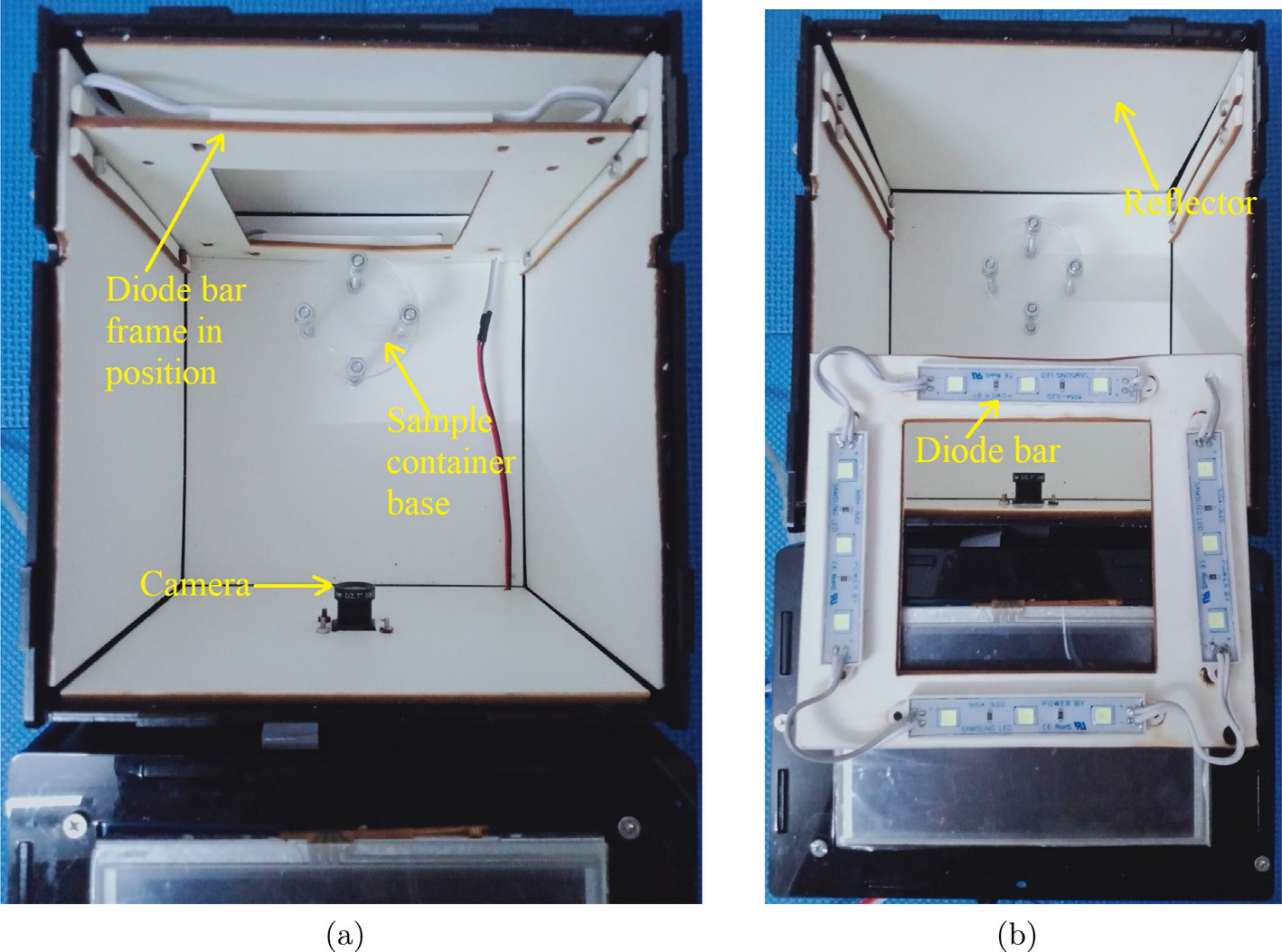
Arrangement inside the designed dark box.

Let it first describe the dark box as it plays a critical role in the design. The dark box contains a sample container 1 that is plastic-transparent and has cylinder shape with a diameter of 3.5 cm and a height of 6 cm. In the measuring mode, the microalgae solution is continuously pumped into the sample container through a 3 mm diameter pipe and then refluxed back to the culture tank. A low-cost Raspberry Pi camera is installed at one of the dark box walls. At a predefined sampling frequency, the camera constantly takes photos of algal microorganisms running through the plastic-transparent sample container 1. The dark box has dimensions of 18 cm × 16.5 cm × 14.5 cm, and the idea to design the box in the equipment is to prevent all external disturbances caused by uncertain natural light influencing on quality of the captured images. In order to provide light inside the box for operations of the camera, an artificial light source was designed by 4 aluminium white light emitting diode (LED) bars forming a square shape as demonstrated in Fig. 3. The bar has a LED chip with surface mounted diodes (SMD) of 5054. There are 3 white diodes equidistantly arranged on each 7.5 cm bar. The white diode can generate colorless daylight at 6500 K color temperature, where its luminous flux is in a range of 20–24 lm. Internal surface of the dark box is covered by smooth-textured white fomex limiting any light reflection and refraction in the box.

One issue to be noted is that if the LED bars are installed facing directly to the sample container, it causes reflection, refraction and high contrast, which leads to inaccurate representation of microalgal density in images. Thus, it was proposed to exploit the bounce technique, a well-known method in photography [17]. To implement the bounce technique in the proposed system, one first designed a reflector deployed at one wall of the dark box. The LED bars were then installed facing directly to the reflector as can be seen in Fig. 2. The arrangement enables the dark box to generate a soft and uniformly distributed light in it, which minimizes phenomena of contrast, shadow, reflection and refraction on the sample container, guaranteeing high quality of the captured images. It is also noticed that distances from the camera lens to the sample container and from the sample container to the artificial light source are about 8 cm and 1.5 cm, respectively.

After being taken by the camera at resolution of 600 × 800 pixels, the images are sent to the image processor, which is implemented on a Raspberry Pi 3 B + for preprocessing and training a learning model [18], [19]. The model is then utilized to predict density of microalgae presenting in a specific microalgal image. The predicted microalgae density is finally used to control the nutrient supplement as well as schedule the harvesting and forwarded to a 7-inch WaveShare HMI for real-time observations.

### 2.2 pH and DO monitoring

Two other environmental parameters including pH and DO also plays a very critical role in the growth rate and productivity of microalgae [20], [21], [22]. For instance, most of microalgal species grow in the culturing environment with pH under 9.5 [20]. Hence, it is crucial to maintain this level in the microalgae chamber. On the other hand, if carbon dioxide in a cultivation medium above $31m.g^{-3}$, biomass productivity of microalgae starts reducing. That is, a predefined level of DO in the culture environment is required to be controlled in order to mitigate any ${\mathrm{CO}}_{2}$ increment.

**Fig. 4:**
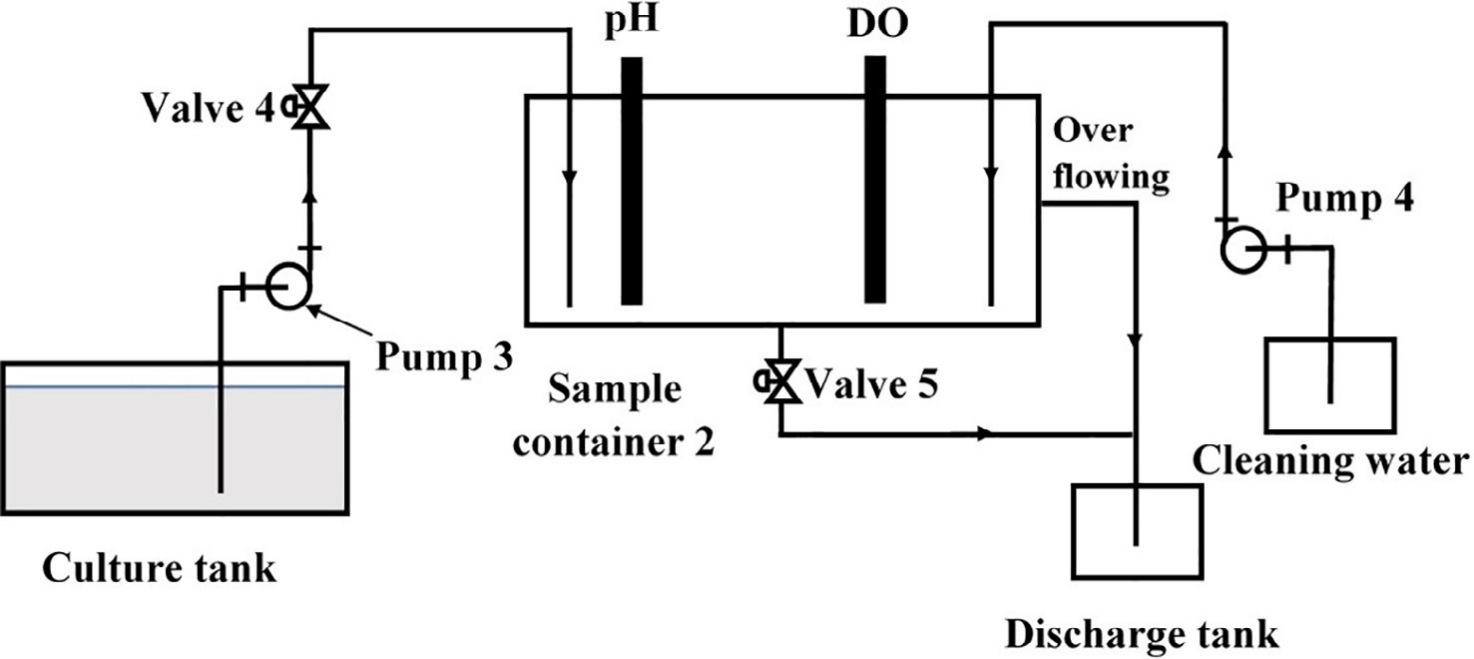
A schematic diagram of monitoring pH and DO.

To continuously and effectively monitor both pH and DO in the microalgae solution, a measuring equipment was designed as demonstrated in Fig. 1. The schematic diagram of this device is presented in Fig. 4. It can be clearly seen that both the pH and DO sensors are deployed in the sample container 2. In the measurement mode, the microalgae solution is pumped from the culture tank to the sample container 2 by Pump 3 through Valve 4. After finishing taking measurements, the sample solution is discharged through Valve 5, and fresh water is pumped into the container by Pump 4. In this design, the Kamoer peristaltic pump was chosen for Pump 3 and Pump 4 while the PURO-XD-12VDC solenoid valve was chosen for Valve 4 and Valve 5, respectively.

The raw measurements taken by the E-201-C pH and DFRobot DO sensors are sent to an Arduino Nano to interpret the real pH and DO values in the culture tank, which can be employed to control those parameters and display on the 7-inch WaveShare HMI for observations.

## 3 Design files summary

This section provides brief summaries of the design files that were developed for the monitoring system. All the files are uploaded to a repository and can be accessed in this link. However, the files are also available with the article as presented below. It is noted that only the design files relevant to the monitoring system including the microalgae density and pH and DO measurement units are provided. The other design files in the continuous culture model such as the culture tank, discharge tank, air and stirring mechanisms are beyond scope of this paper.

### 3.1 Microalgal density measurement unit

#### 3.1.1 Dark box

**Fig. 5:**
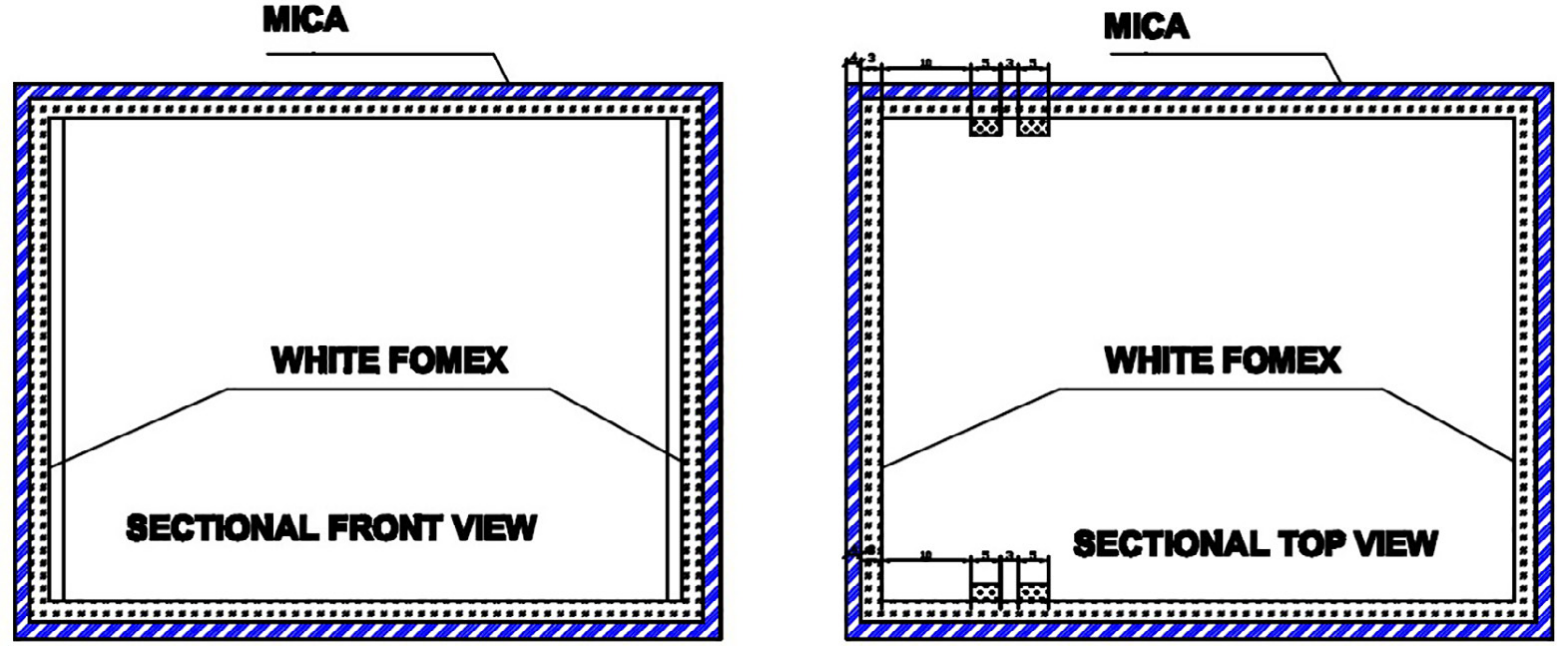
The layer views of the dark box.

**Fig. 6:**
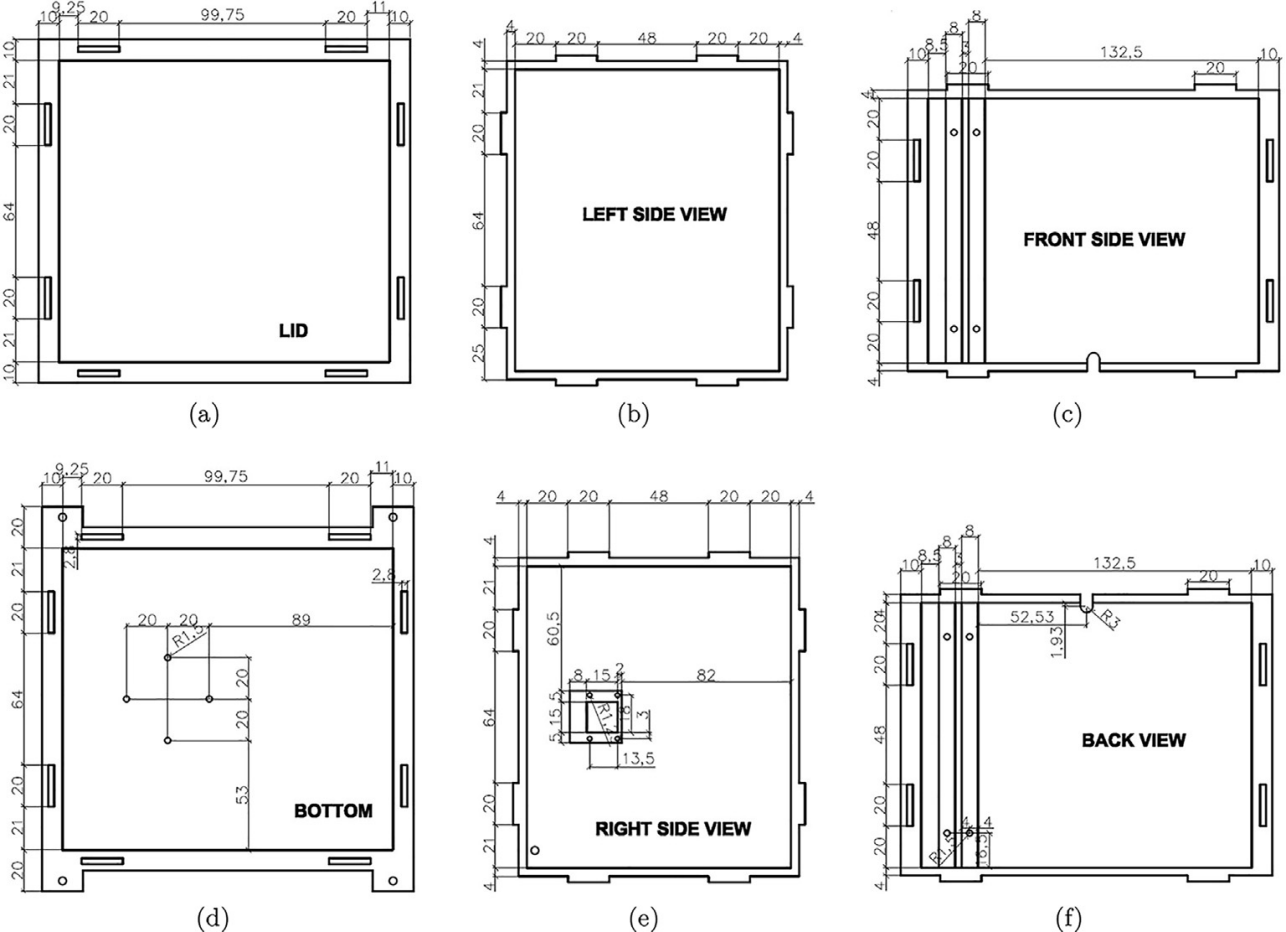
The six views of the designed dark box.

As discussed in Section 2, the first component in the microalgae density measurement unit is the dark box. The box has two layers as can be seen in Fig. 5 where the external layer is made of mica while the internal one is made of smooth-textured white fomex to minimize any light reflection and refraction. The dimensions of the box are detailed in Fig. 6.

**Fig. 7:**
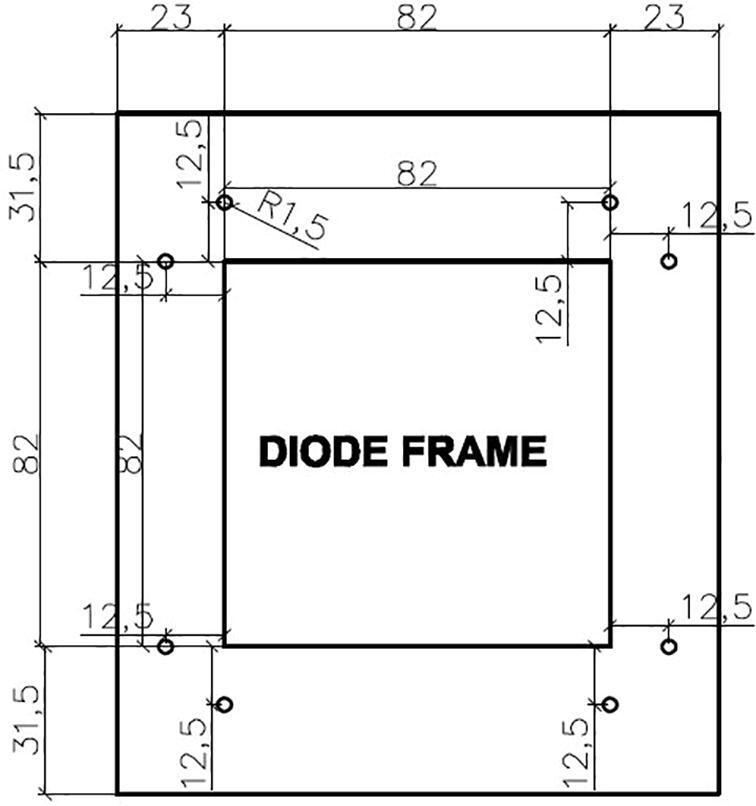
The design of the diode frame.

Another component in the dark box is the artificial light that is generated by the LED bars. In the design, the LED bars are mounted on a frame as illustrated in Fig. 7.

#### 3.1.2 Image processor and HMI

**Fig. 8:**
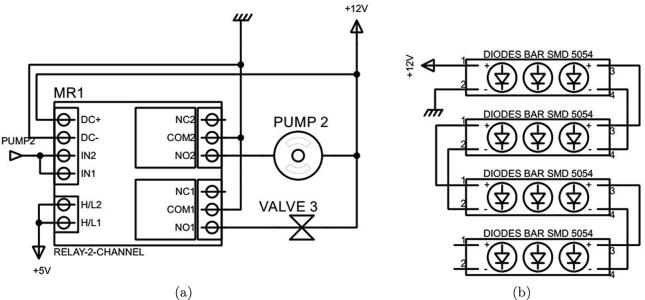
The circuit connections of the image processor and HMI.

**Fig. 9:**
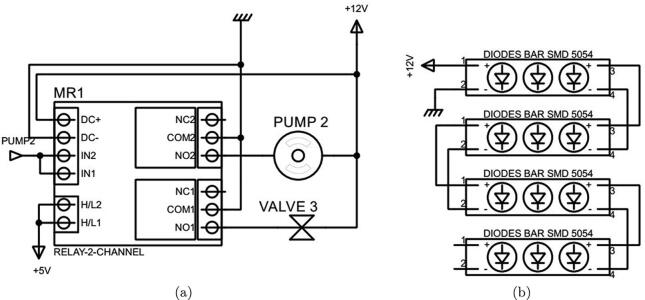
The circuit connections of (a) Pump 2 and Valve 3, and (b) the artificial light source.

In the design, a Raspberry Pi 3 B + was used as an image processor where the raw images captured by the camera are processed. A learning model is also trained on this Raspberry Pi 3 B + given the microalgae images, which is then utilized to predict density of the microalgae. As can be seen in Fig. 8a, the processor is connected to a low-cost Raspberry Pi OV5647 camera. The microalgae density, after being computed by the processor, is displayed on the HMI as shown in Fig. 8b. Morevover, the Raspberry Pi 3 B + is employed to control Pump 2, where the control signal is sent to the power actuator circuit in Fig. 9a. It is noted that two buttons BT1 and BT2 were also designed as can be seen in Fig. 8a to manually control Pump 2 and the camera.

#### 3.1.3 Power gain and artificial light

As discussed in Section 2, in this design a Kamoer peristaltic pump was chosen for Pump 2 and a PURO-XD-12VDC solenoid valve as selected for Valve 3. These both actuators are run at  + 12 VDC. Therefore, a 12 V - 2 channels optocoupler relay was selected to supply power to them, as can be seen in Fig. 9a. The optocoupler also isolates the processor from the  + 12 V source. It is noted that whenever Pump 2 operates, Valve 3 is opened simultaneously.

The artificial light is generated by four aluminum white LED bars forming a square shape. Each 7.5-cm bar has three surface-mounted diodes of 5054, which operates at  + 12 VDC, as illustrated in Fig. 9b.

### 3.2 pH and DO measurement unit

#### 3.2.1 Sample container 2

**Fig. 10:**
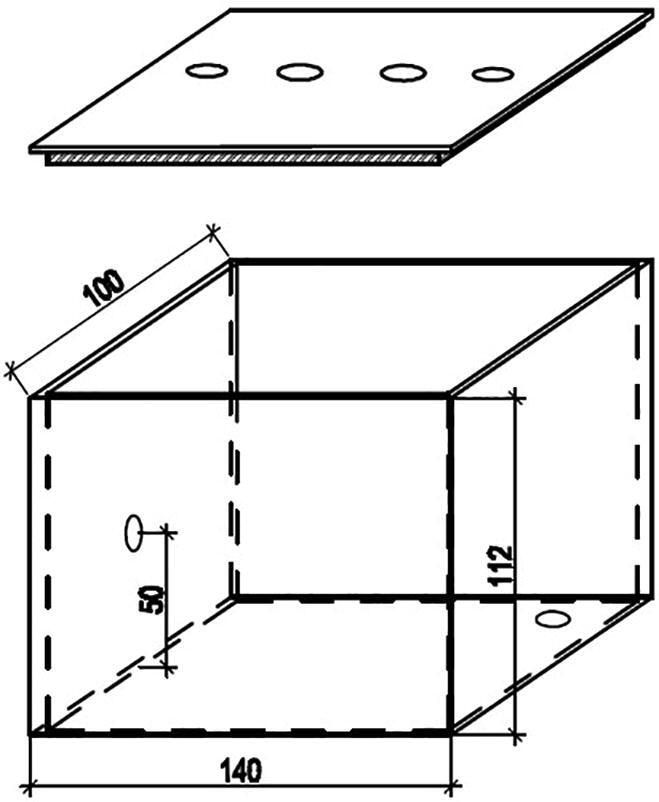
Overview of the sample container 2.

**Fig. 11:**
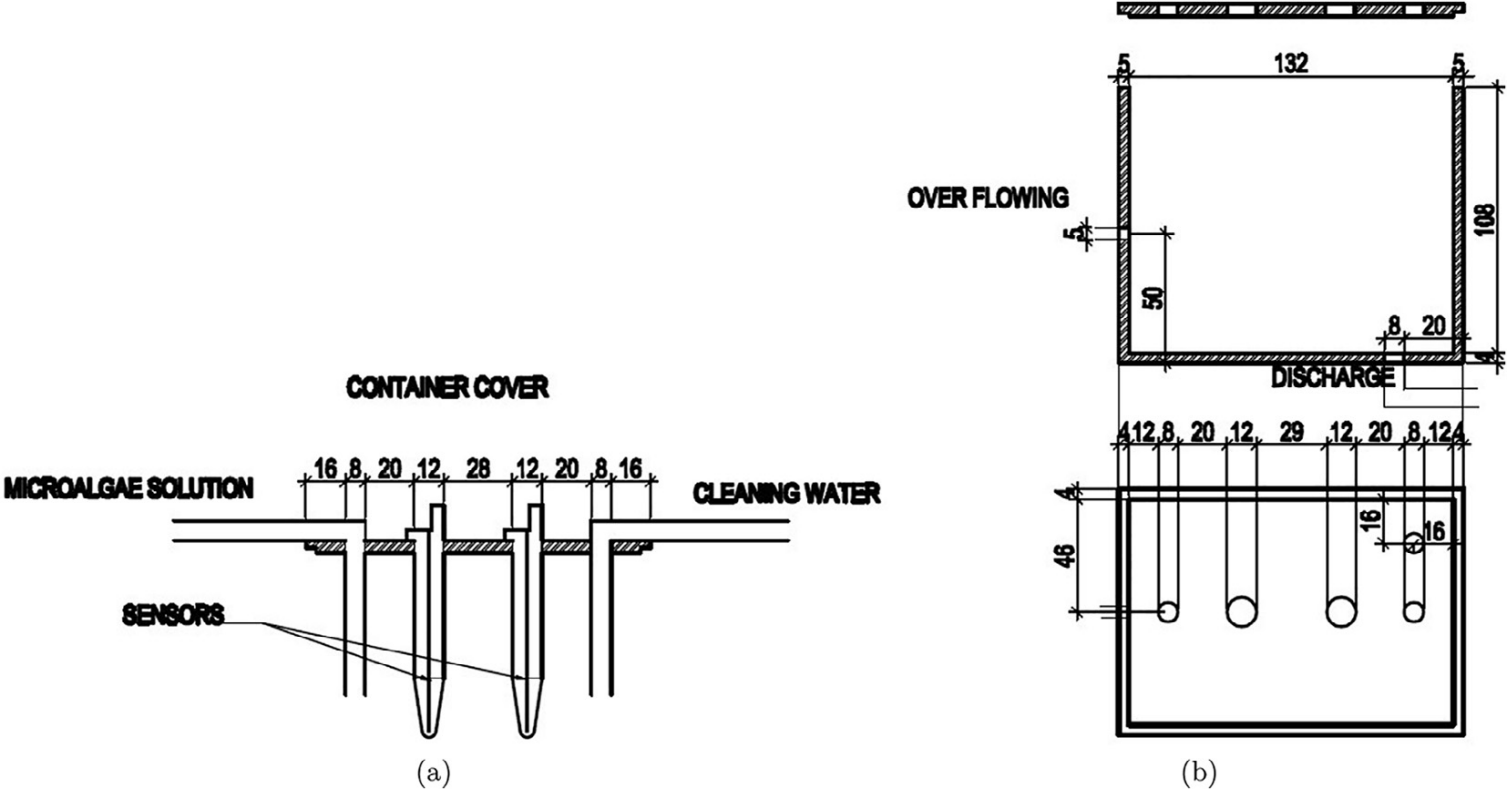
The design details of the sample container 2.

The first component in the pH and DO measurement unit is the sample container 2, whose dimension overview is shown in Fig. 10. In the design, mica material was used to fabricate the container. Other design details including the sensor positions, the solution and cleaning water tube inlets, the discharge tube outlet and the overflowing position are sketched in Fig. 11.

#### 3.2.2 pH and DO sensors

**Fig. 12:**
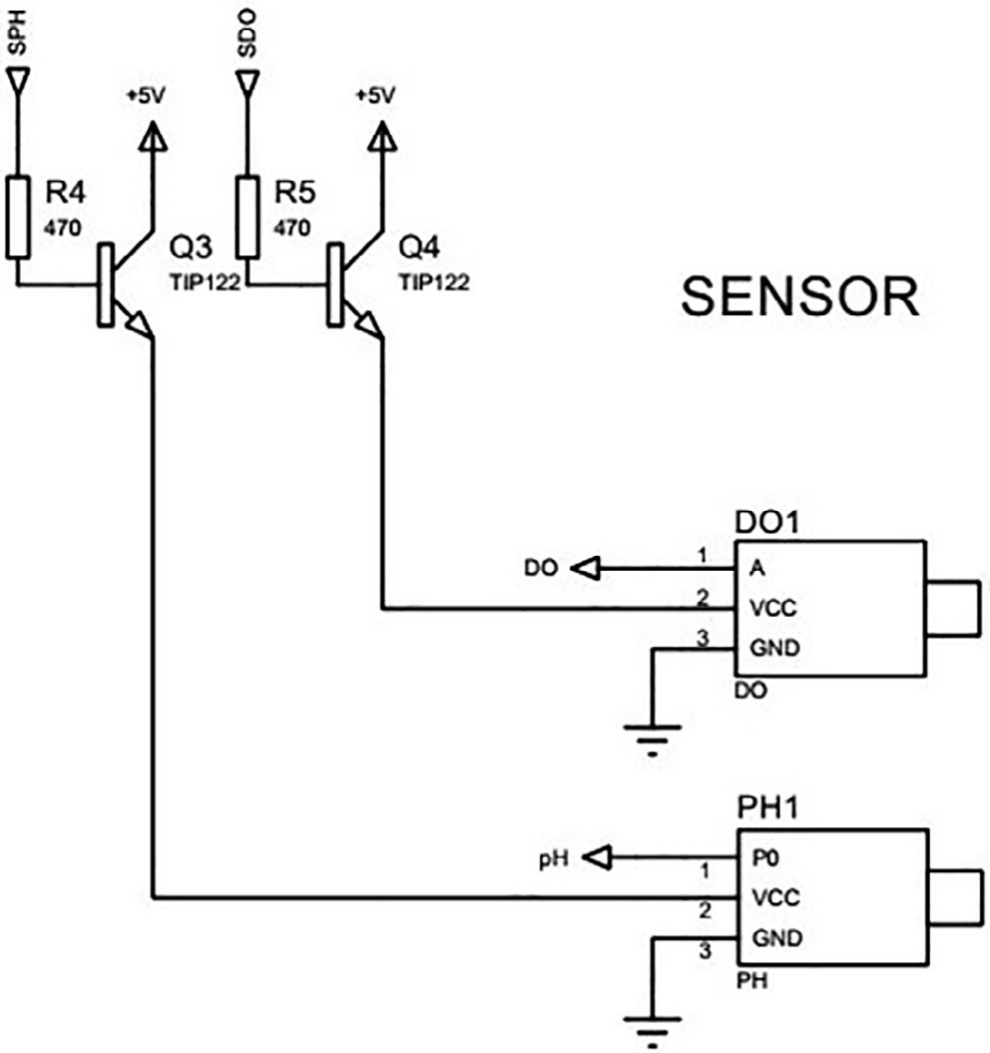
The circuit connection of pH and DO sensors.

**Fig. 13:**
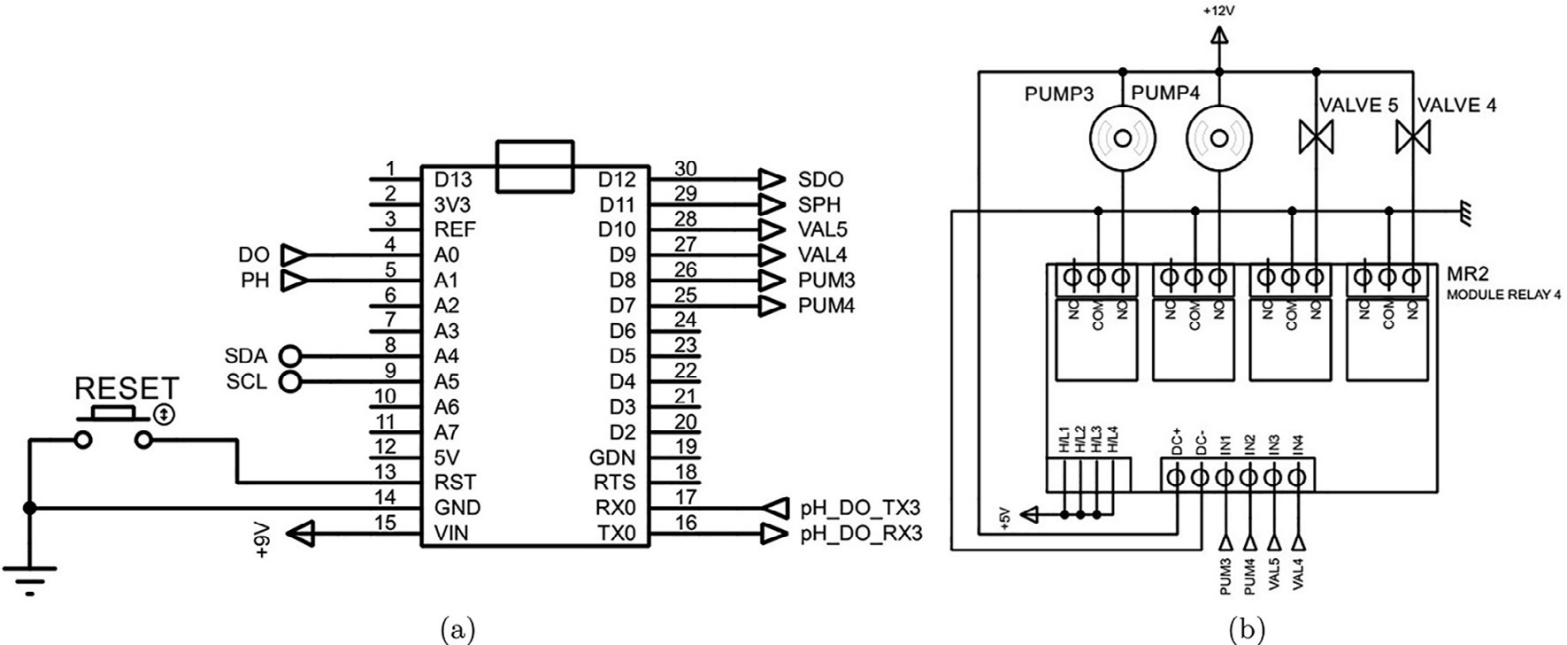
The processor and actuator circuits in the pH and DO measurement unit.

The low cost E-201-C pH and DFRobot DO sensors are chosen in this study to measure pH and DO parameters. The measurement in these sensors is controlled by an Arduino Nano microcontroller as can be seen in Fig. 12, Fig. 13a. In other words, the microcontroller can switch the sensors on or off through two TIP122 transitors in Fig. 12. And the sensor output signals are directly sent to the microcontroller.

#### 3.2.3 Sensor signal interpretation and actuator control

The pH and DO sensor signals are received and interpreted by the Arduino microcontroller to compute the pH and DO quantities in the culture tank, as demonstrated in Fig. 13a. Moreover, the microcontroller is also used to control Pump4, Pump 5, Valve 3 and Valve 5 through a 12 V - 4 channels optocoupler relay during the measurement period as illustrated in Fig. 13b.

### 3.3 Software and firmware files

**Fig. 14:**
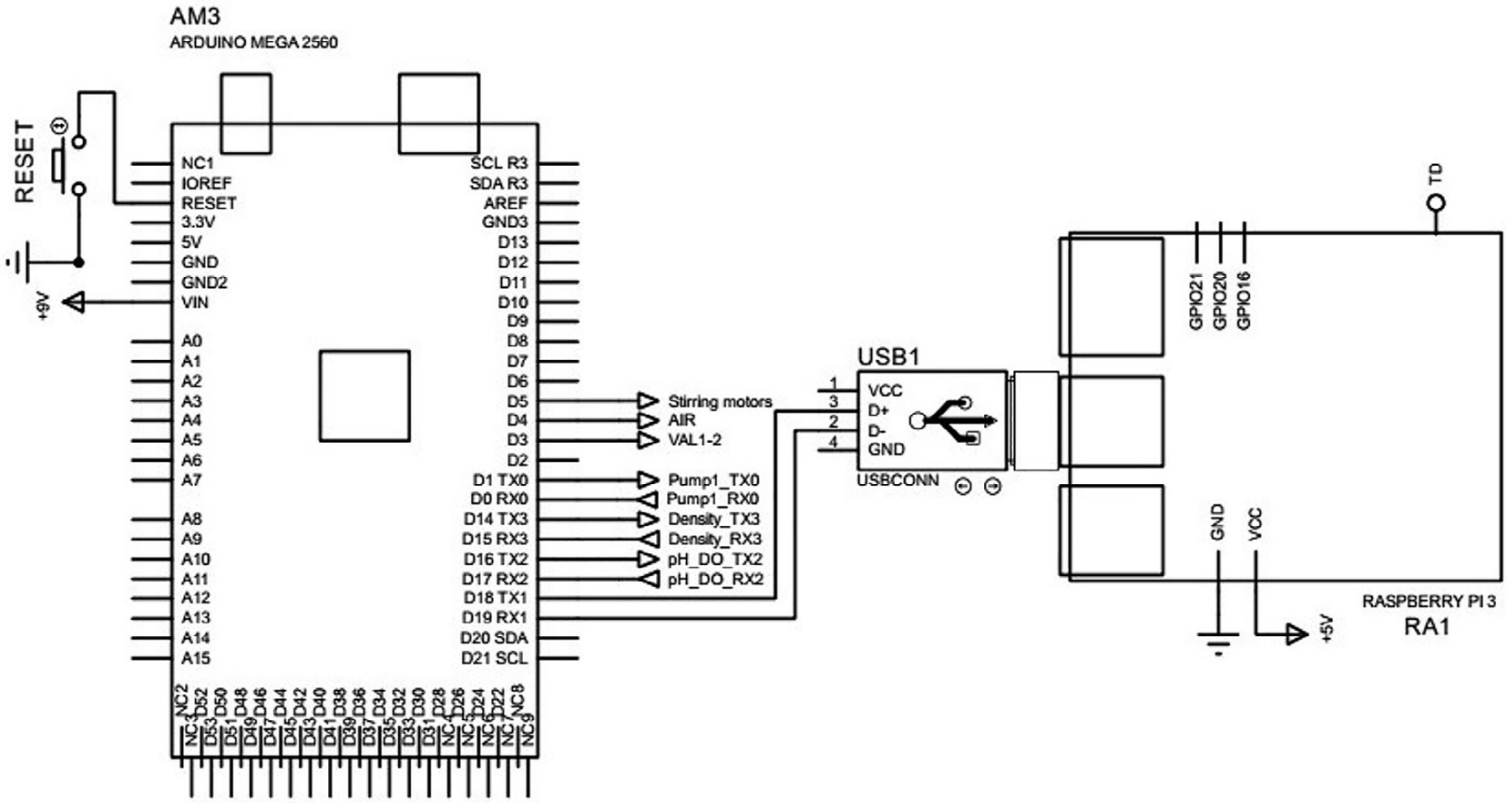
The central control unit.

Apart from the CAD and electronic circuit files aforesaid, there are also some software and firmware files used to run on the Raspberry Pi 3 Model B + and upload to the Arduino microcontrollers for the control and calculations. For instance, the file *Central control unit.ino* can be used to upload to the Arduino Mega 2560 in the central control unit as shown in Fig. 14 to set up when the measurements can be taken. Since the Arduino Mega 2560 is connected to both the Raspberry Pi 3 Model B + and Arduino Nano in Fig. 13, the program is also employed to read the measurements of pH, DO and microalgal density for other purposes such as recording, controlling other actuators in the whole cultivation system or displaying for real-time observations. The other file *pH DO measurement.ino* is uploaded to the Arduino Nano as depicted in Fig. 13 for calibrating the DO sensor and interpreting the pH and DO measurements from the raw sensor signals.

On the other hand, the files *Automatic mode interface.ui, Microalgae graph interface.ui, pH graph interface.ui and DO graph interface.ui* are run on PyQt5 and Python installed on the Raspberry Pi 3 Model B + to compute and display the microalgal density, pH and DO on the HMI device. The file *Main.py* is run on the Raspberry Pi 3 Model B + and utilized to predict density of microalgae given their captured images. It is noted that while the microalgal density values are computed within the Raspberry Pi 3 Model B+, the pH and DO measurements are read by Arduino Mega 2560 from the Arduino Nano and transmitted to the Raspberry Pi 3 Model B + through the communication as can be seen in Fig. 14. The measurements are sent to the HMI from the Raspberry Pi 3 Model B + as shown in Fig. 8.

All these software and firmware files are also uploaded to the repository.

## 4 Bill of materials summary

Bills of materials that were purchased for the system are summarized in an Excel spreadsheet, which can be found in this link.

## 5 Build instructions

This section provides information of the required components and step-by-step instructions to build two measurement units to monitor the microalgal density and pH and DO parameters in an algae culture environment.

### 5.1 Building microalgal density measurement unit

#### 5.1.1 Building the sample container 1

*Required components:* 02 80 mm Φ4 silicone pipes, 02 elbows $90^{o}\Phi 4$, 02 male couplings 3–3, 01 55 mm Φ4 silicone pipe, 02 30 mm Φ4 silicone pipes, 02 reducers 6–4, silicone sealant, 01 sample container.

*Building steps:*

**Fig. 15:**
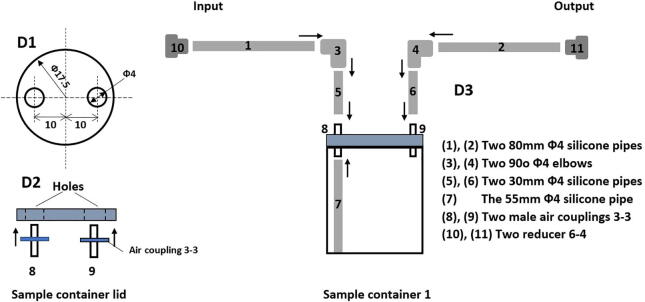
Building the sample container 1.
• Insert 02 male couplings into the two holes on the lid and fix them with silicone sealant as demonstrated in Fig. 15 D2.
• Assemble 01 80 mm Φ4 silicone pipe, 01 elbows $90^{o}\Phi 4$ and 01 30 mm Φ4 silicone pipe to make a couple of the L shape pipes as illustrated in Fig. 15 D3.
• Connect these two L shape pipes to two male couplings 3–3 on top of the container lid.

• Drill 02 Φ3 holes on the container lid as shown in Fig. 15 D1.

#### 5.1.2 Building the dark box

*Required components:* 01 dark box, 01 diode frame, 01 diode SMDs 5054 bar (1000 mm), 300 mm 24D Φ1.6 wire, 01 Raspberry Pi camera OV5647, the sample container 1 and other electric cables.

*Building steps:*

**Fig. 16:**
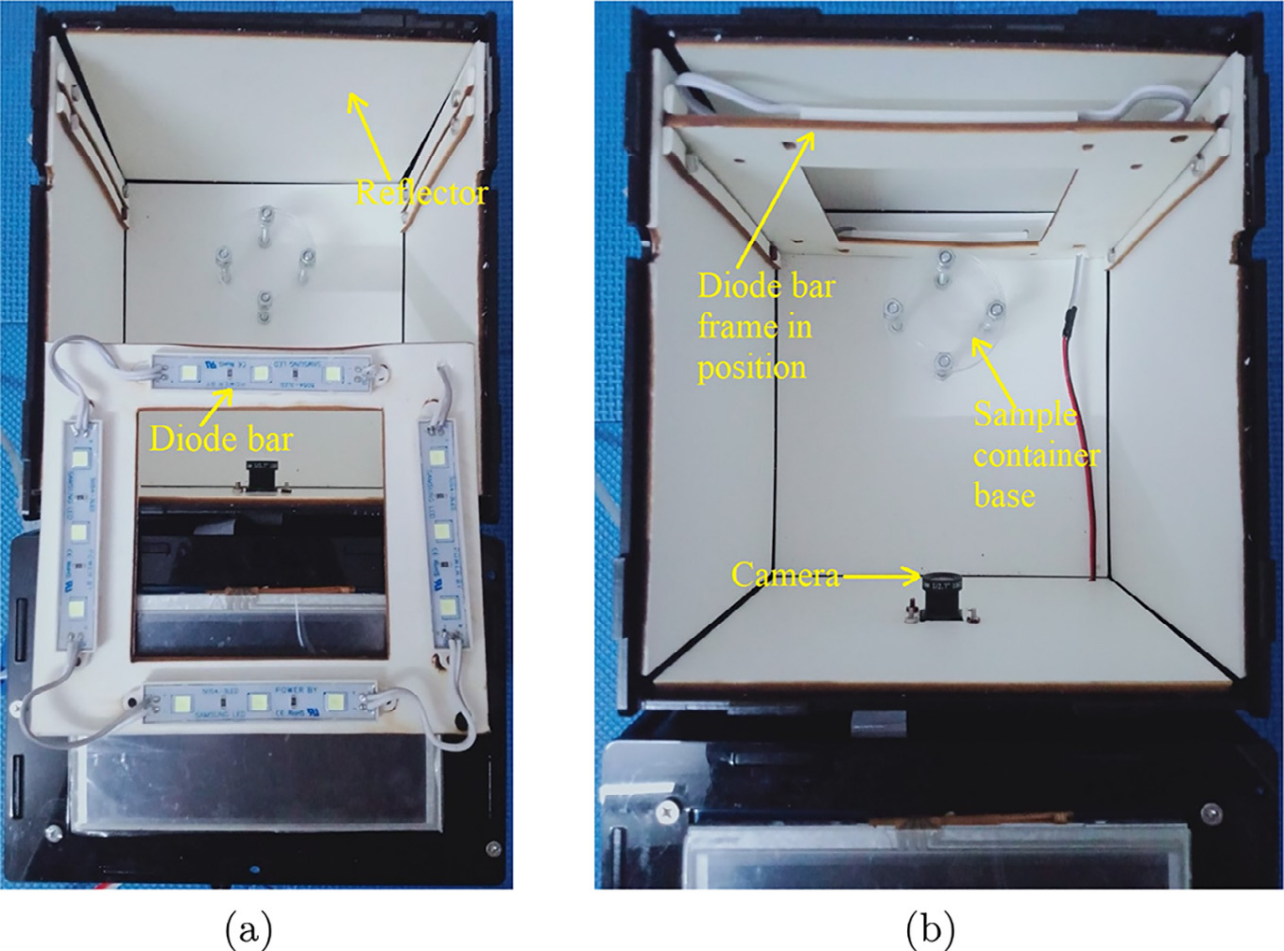
Installation of the dark box.
• Connect the diode segments together and then to the power as shown in Fig. 9b.
• Connect the Raspberry Pi camera OV5647 to the Raspberry Pi 3 Model B + as illustrated in Fig. 8a.
• Install the sample container 1 to the sample container base as shown in Fig. 16b.
• Install the camera and diode frame to the dark box given the information provided in Section 3.
• Cover the lid of the dark box.

• Fabricate the dark box and diode frame using the design information in Section 3.1.1.
• Cut 04 75 mm diode segments, where each segment has 3 diodes, from the diode SMDs 5054 bar.
• Install 04 75 mm diode SMDs 5054 bars on the diode frame. The results can be seen in Fig. 16a.

#### 5.1.3 Assembling the image processor, microcontroler and HMI

*Required components:* 01 Raspberry Pi 3 Model B + with 01 SanDisk microSD Ultra 32 GB Class 10 SDSQUNR-032G-GN3MN (Memory), 01 7 inch WaveShare HMI, 01 Arduino Mega 2560, 01 USB/UART cable CP2102, 03 buttons, 24D Φ1.6 wire and other electric cables.

*Building steps:*

• Connect the Raspberry Pi 3 Model B + to the Raspberry Pi camera OV5647 as shown in Fig. 8.
• Connect the Raspberry Pi 3 Model B + to the 7 inch WaveShare HMI as depicted in Fig. 8.
• Connect the Raspberry Pi 3 Model B + to the Arduino Mega 2560 through the USB/UART cable CP2102 as can be seen in Fig. 14.
• Connect two buttons to the Raspberry Pi 3 Model B + as demonstrated in Fig. 8.
• Upload the file *Central control unit.ino* to the Arduino Mega 2560.
• Install PyQt5 and Python on Raspberry Pi 3 Model B+.
• Upload the files *Automatic mode interface.ui, Microalgae graph interface.ui, pH graph interface.ui and DO graph interface.ui* and *Main.py* to the Raspberry Pi 3 Model B+.
• Connect power to the image processor, microcontroler and HMI.

#### 5.1.4 Assembling Pump 2 and Valve 3 to the dark box

*Required components:* 01 Kamoer 12VDC peristaltic pump, 01 PURO-XD - 12VDC solenoid valve, 01 12 V 2-channel relay module with optocoupler H/L level trigger, 01 100 mm silicon Φ10 pipe, 01 6300 mm silicon Φ6 pipe, 02 reducers 6–4, 02 reducers 10–6 and other electric cables.

*Building steps:*

**Fig. 17:**
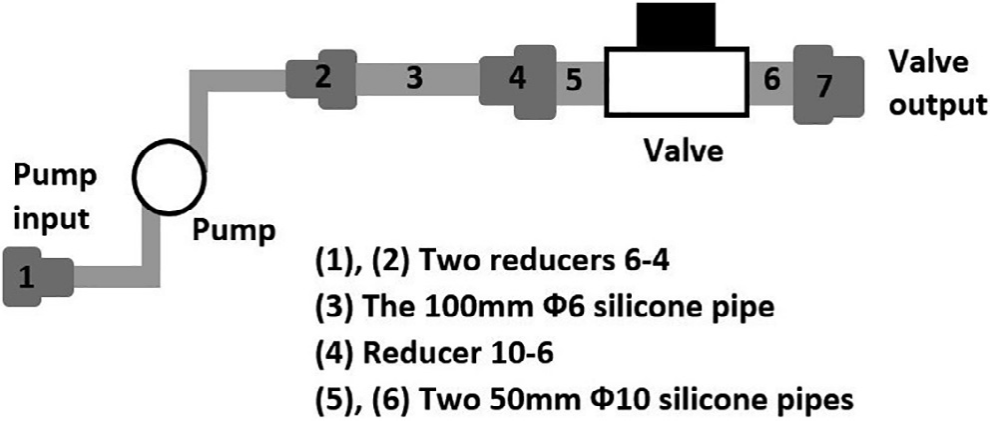
Building Pump 2 and Valve 3 to the dark box.
• Connect one end of one 2000 mm Φ6 silicone pipe to the pump input, the other end is deployed in the culture tank.
• Use another 2000 mm Φ6 silicone pipe to connect the valve output to the dark box.
• Use the third 2000 mm Φ6 silicone pipe to connect the output of the dark box to the culture tank.
• Connect Pump 2 and Valve 3 to the Raspberry Pi 3 Model B + through the 12 V 2-channel relay module with optocoupler H/L level trigger as demonstrated in Fig. 9a.
• Connect Pump 2 and Valve 3 to a 12 V power supply.

• Cut the 100 mm Φ10 silicone pipe into 02 50 mm segments.
• Cut the 6300 mm Φ6 silicone pipe into 05 segments of 02 2000 mm, 01 200 mm and 01 100 mm, respectively.
• Assemble the pipe segments, reducers, Kamoer 12VDC peristaltic pump and 01 PURO-XD - 12VDC solenoid valve to form the solution supply mechanism as can be seen in Fig. 17.

### 5.2 Building pH and DO measurement unit

#### 5.2.1 Sensing platform

*Required components:* 04 150 mm Φ8 silicone pipes, 02 60 mm Φ8 silicone pipes, 02 40 mm Φ8 silicone pipes, 06 elbows $90^{o}\Phi 8$, 01 reducer 10–8, 01 coupling 8–8, 04 reducers 6–4, 1 100 mm Φ6 silicone pipe, 01 reducer 10–6, 02 50 mm Φ10 silicone pipes, 01 reducer 10–8, 02 Kamoer 12VDC peristaltic pumps, 02 PURO-XD - 12VDC solenoid valve, 01 reducer 8–6, 01 40 mm Φ6 silicone pipe, 02 2000 mm Φ6 silicone pipes, 02 1500 mm Φ8 silicone pipes, 01 cleaning water tank, 01 discharge tank and the sample container 2.

*Building steps:*

**Fig. 18:**
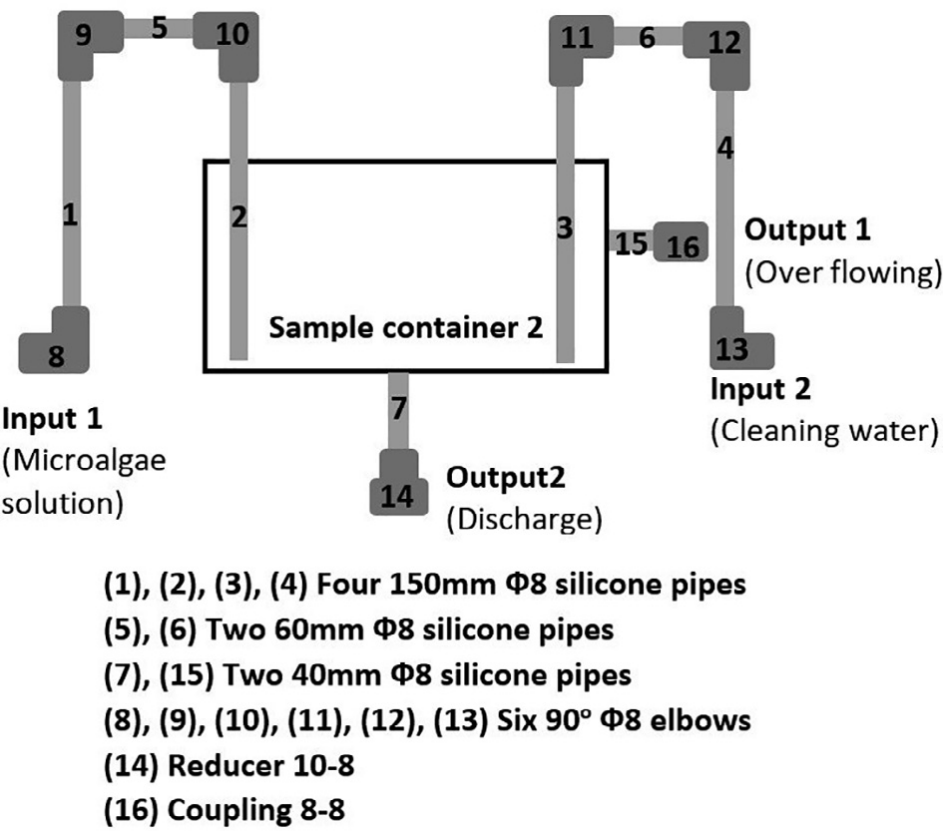
Building the sample container 2.
• Following the diagram in Fig. 17, assemble the given pipes, pump, valve and reducers to form the solution supply system to the sample container 2.
• Following the diagram in Fig. 19, assemble the given pipes, pump and reducers to form the cleaning water supply system to the sample container 2.

• Fabricate the sample container 2 using the design information in Section 3.2.1.
• Following the diagram in Fig. 18, assemble the given pipes and reducers to form the supplying and discharging pipes to the sample container 2.

**Fig. 19:**
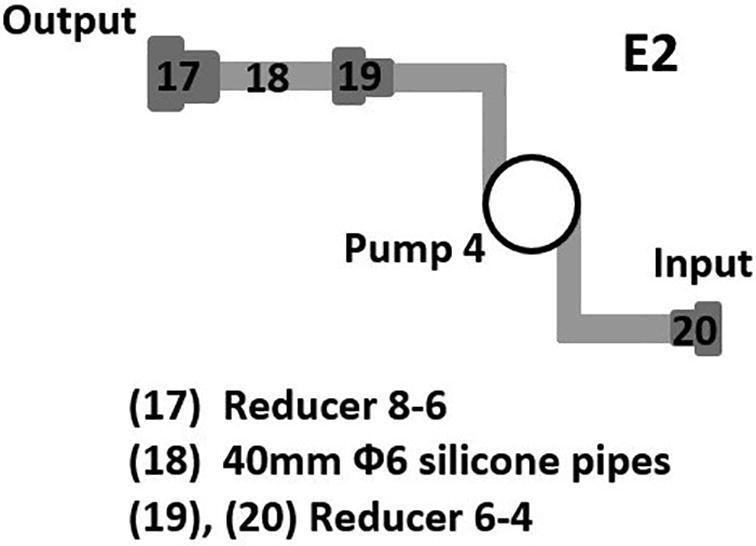
Building the cleaning water supply to the sample container 2.
• Use a 60 mm Φ8 silicone pipe to connect Input 1 in Fig. 18 to Valve output in Fig. 17.
• Use a 2000 mm Φ6 silicone pipe to connect Pump input in Fig. 17 to the culture tank.
• Use another 60 mm Φ8 silicone pipe to connect Input 2 in Fig. 18 to Output in Fig. 19.
• Use another 2000 mm Φ6 silicone pipe to connect Input in Fig. 19 to the cleaning water tank.
• Connect a PURO-XD - 12VDC solenoid valve to Output 2 in Fig. 18. Then use a 1500 mm Φ8 silicone pipe to connect the valve to the discharge tank.
• Use another 1500 mm Φ8 silicone pipe to connect Output 1 in Fig. 18 to the discharge tank.

#### 5.2.2 Assembling the sensors, processor, pumps and valves

*Required components:* 01 Arduino Nano CH340, 01 12 V 4-channel relay modules with optocoupler H/L level trigger, 02 TIP122 transistors, 02 470Ω resistors, 01 Button, 01 2000 mm 24D Φ1.6 wire, 01 E-201-C pH sensor, 01 DFRobot DO sensor and other electric cables.

*Building steps:*

• Connect the sensors to the transistors and resistors as shown in Fig. 12.
• Deploy the sensors in the sensor cages in the sample container 2 as presented in the design in Section 3.2.1.
• Connect the other ends of the resistors in Fig. 12 to the Arduino Nano CH340 in Fig. 13a.
• Connect the Arduino Nano CH340 to the Arduino Mega 2560 in Fig. 14.
• Connect Pump 3, Pump4, Valve 4 and Valve 5 to the Arduino Nano CH340 through the 12 V 4-channel relay module with optocoupler H/L level trigger as demonstrated in Fig. 13b.
• Upload the file *pH DO measurement.ino* to the Arduino Nano CH340.
• Connect power to the sensors, microcontroller, pumps and valves.

## 6 Operation instructions

### 6.1 Calibrations

In order to operate the proposed monitoring system, it is required to calibrate the sensing modules. In fact, the pH, DO and microalgal density sensing modules were already calibrated and incorporated into the programs *pH DO measurement.ino* and *Main.py*, and the monitoring system is ready to be used. Nevertheless, this section provides the calibration procedures for those sensing modules in cases when the monitoring system is reproduced, it can be recalibrated to ensure accuracy of measurements.

#### 6.1.1 pH sensor calibration

**Fig. 20:**
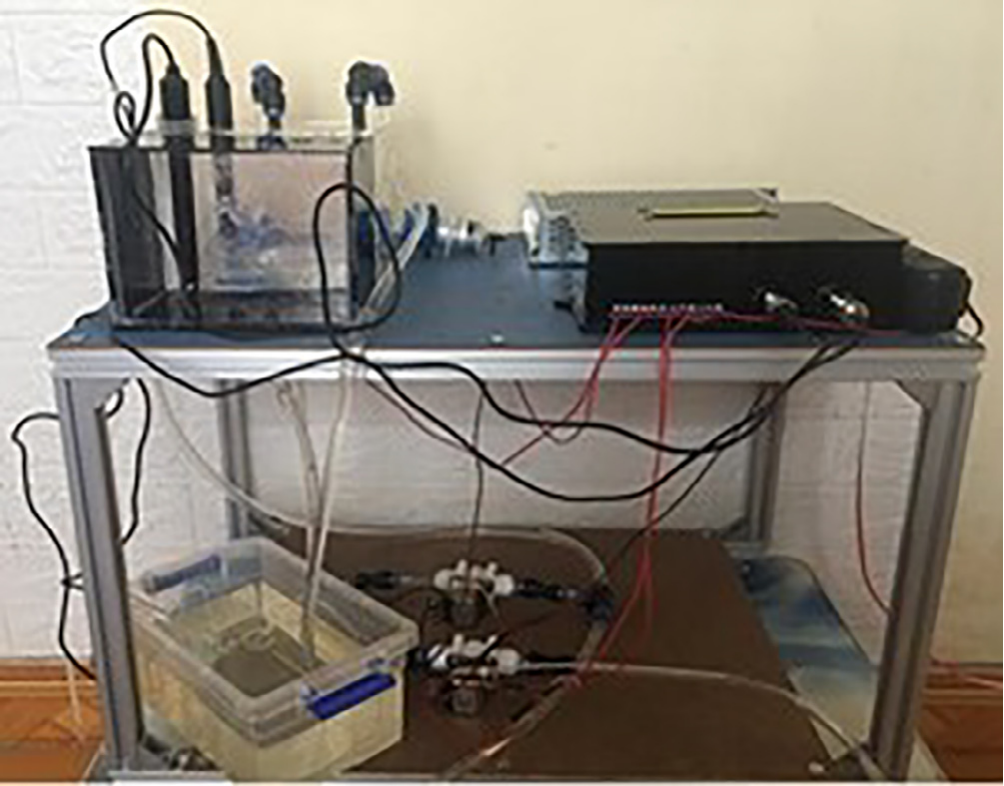
The pH and DO measurement unit.

The setup for calibrating both the pH and DO sensors is demonstrated in Fig. 20. In order to calibrate the pH sensor, the buffer solutions with standard pH values of 4.01, 7.00 and 10.01 were exploited. The sensor probe was sequentially dipped into the buffer solutions. The sensor output voltage was also sequentially recorded by a multimeter. From three pairs of the voltage and pH values, it showed that relationship between the voltage and the pH quantity is quite linear. Therefore, a straight line was fitted through three data points to form a linear equation presenting computation of pH values given the sensor output voltage (Volt) as follows,

(1) pH=-6.0274*Volt+22.236.

**Fig. 21:**
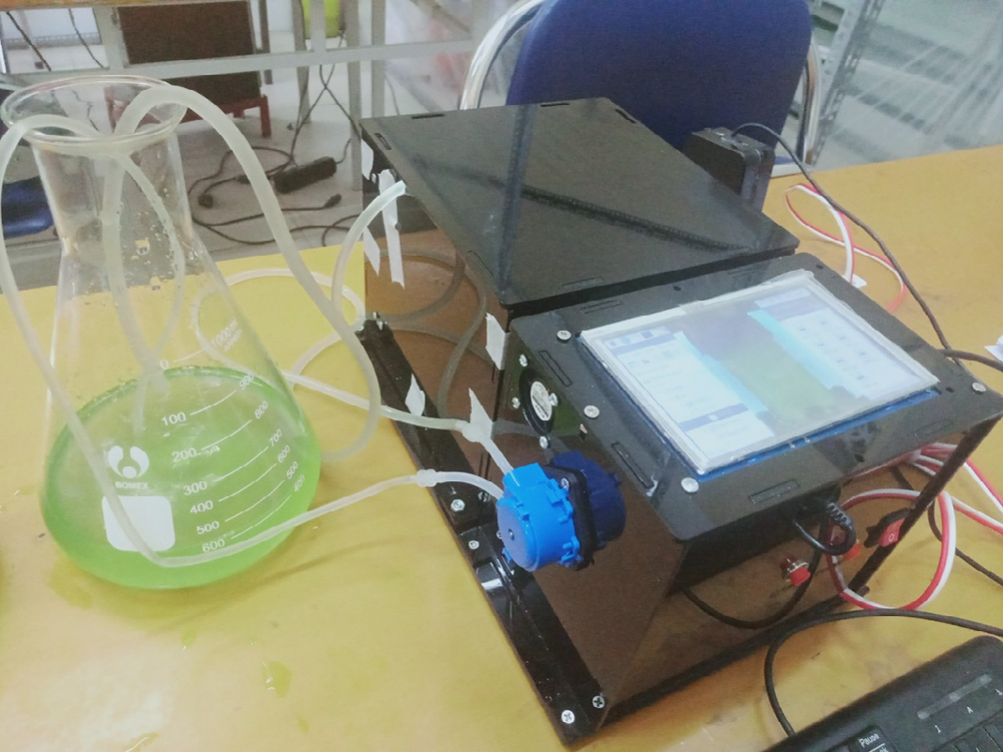
The microalgal density measurement unit.

The Eq. (1) has already been implemented in the program *pH DO measurement.ino*. Fig. 21.

#### 6.1.2 DO sensor calibration

In contrast to the pH sensor, the purified water was used for calibrating the DO sensor. In the first step, an air pump was employed to continuously inflate the water for about 10 min so that the dissolved oxygen is saturated in the water. After the bubbles in the water disappeared, the DO sensor probe was dipped into the water. While holding the sensor inside the water, the water was slowly stirred without generating any new bubbles. A multimeter Was utilized to monitor the sensor output voltage. Once the voltage value is stabilized, a voltage value that is called a saturation point voltage was recorded. In the meantime, DO in the water was also measured by using a high accurate HI8043 DO meter, which is called saturated DO. Hence, DO value in future measurements can be calculated by the following equation given the sensor output voltage (Volt).

(2) $$\mathrm{DO}=\frac{8.81}{1.16}\mathrm{Volt}\text{.}$$

The Eq. (2) has also been implemented in the program *pH DO measurement.ino*.

#### 6.1.3 Microalgal density measurement calibration

In order to calibrate the microalgal density measurement unit, the manual direct microscopic method was employed. The microalgae solution Was first filled into the sample container 1, and an image of the microalgae was captured using the proposed monitoring system. A sample of 1 ml the same microalgal solution was then mixed with a drop of 1% - 2% Lugol’s iodine. By utilizing a micropipette, 3.2 μl of the mixed solution was filled in a haemocytometer chamber (Fuchs Rosenthal). After about 1 min, all the microalgal cells were evenly distributed in the chamber. The haemocytometer chamber was then placed under a bright light microscope (Zeiss, USA) at ×10 objective. And the microalgae in 4/16 squares were counted. That is, the accurate density of the microalgae in the examined solution was known. This procedure Was repeated 129 times for 129 different microalgal solutions, which resulted in 129 microalgal images and 129 microalgal density values. Features from the images were extracted and then utilized to build a learning model, where the model inputs are the image features while the model output is the microalgal density value. The learned model has already been implemented in the program *Main.py*. In future measurements, once an image of microalgae is captured, the features of the image can be extracted and input into the learned model. The model then predicts the density of the microalgae presenting in the image.

### 6.2 Operation guideline

After fabricating, building and assembling the measurement units as discussed in Section 5, operate the monitoring system as follows.

• Deploy the units to the continuous culture system as can be seen in Fig. 1.
• Supply power to the microcontrollers, Raspberry Pi 3 Model B+, camera, sensors, artificial light source, pumps and valves.
• Turn on the Arduino Mega 2560, Arduino Nano CH340, Raspberry Pi 3 Model B+, camera, HMI.
• Run the program *Central control unit.ino* on the the Arduino Mega 2560.
• Run the program *pH DO measurement.ino* on the Arduino Nano CH340.
• Run the program *Main.py* on the Raspberry Pi 3 Model B+.
• Run the program *Automatic mode interface.ui* on the Raspberry Pi 3 Model B+, then the interface will appear on the HMI with the real-time measurements for observations.
• After each measurement, the electrodes of the pH and DO sensors should be cleaned by the deionized water to ensure high accuracy in next measurements.

### 6.3 Automatic program explanation

Though the monitoring system is programmed to run in an automatic mode, this section provides explanations of how the programs run in a step-by-step manner for better operations.

#### 6.3.1 How the microalgal density measurement unit works

**Fig. 22:**
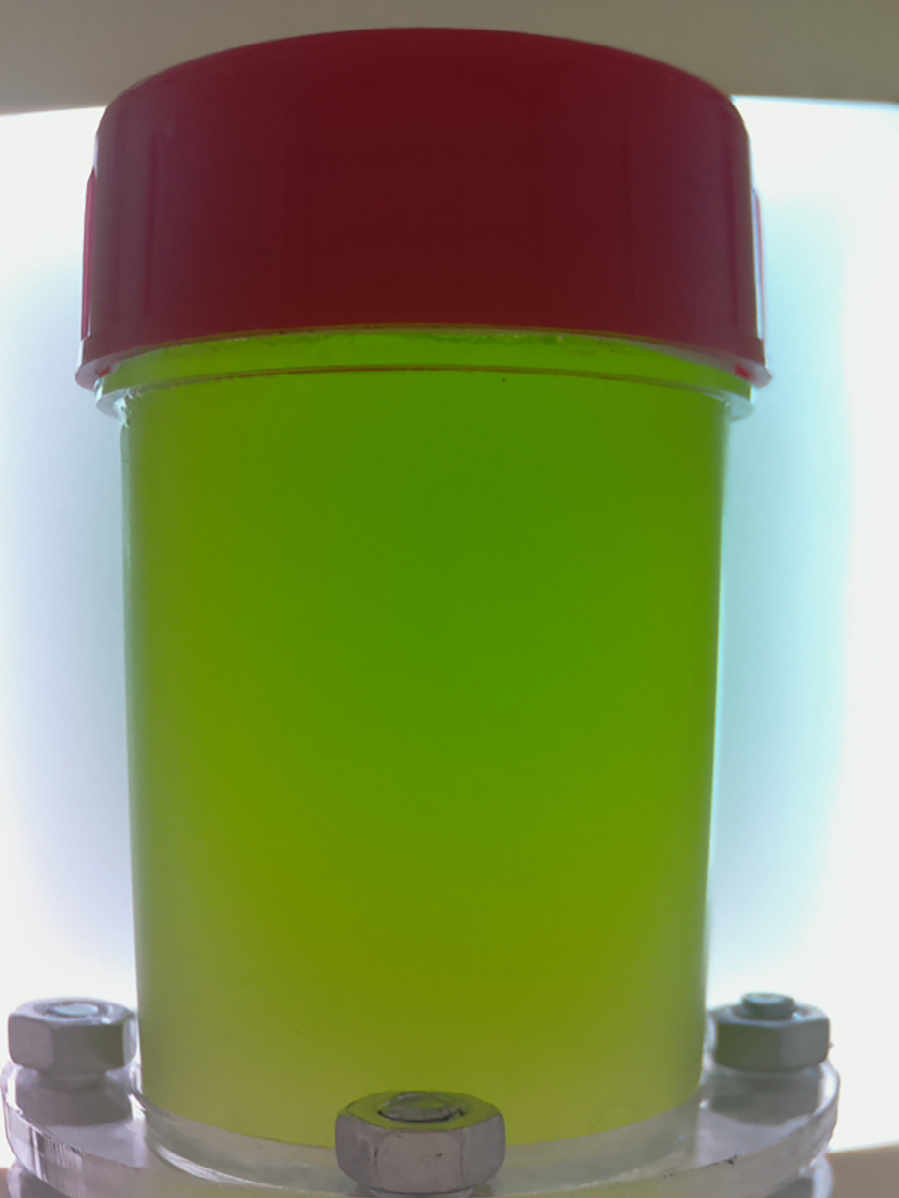
An image of microalgae captured by the camera.

Once a command is sent out from the program *Central control unit.ino* that requires the microalgal density measurement unit to measure density of microalgae, both Pump 2 and Valve 3 are turned on. The microalgae solution is pumped into the sample container 1. When the sample container 1 is filled up by the algal solution, Pump 2 is turned off and Valve 3 is closed, the camera captures an image of the sample container 1 as can be seen in Fig. 22. The image is then sent to the Raspberry Pi 3 Model B + for preprocessing. Features are also extracted on each image, which are then input into a learning model [18], [19] to predict density of the microalgae captured in the image. Processing the microalgae image and predicting the microalgal density are manipulated by the program *Main.py*. The predicted results are sent to the HMI for displaying and the Arduino Mega 2560 for other purposes.

#### 6.3.2 How the pH and DO measurement unit works

Similar to the microalgal density measurement unit, when the pH and DO measurements are requested, the microalgae solution is pumped from the culture tank until the sample container 2 is full. The pH and DO sensors are then turned on to take measurements where the sensor output signals are transmitted to the Arduino Nano CH340 for further processing. The raw signals from the sensors are interpreted by the program *pH DO measurement.ino* on the Arduino Nano CH340. The interpreted results are sent to the Arduino Mega 2560 for displaying and other purposes. Once the measurements are completed, the solution is discharged through Valve 5 and the cleaning water is pumped into the sample container 2 for cleaning the sensor electrodes.

## 7 Validation and characterization

**Fig. 23:**
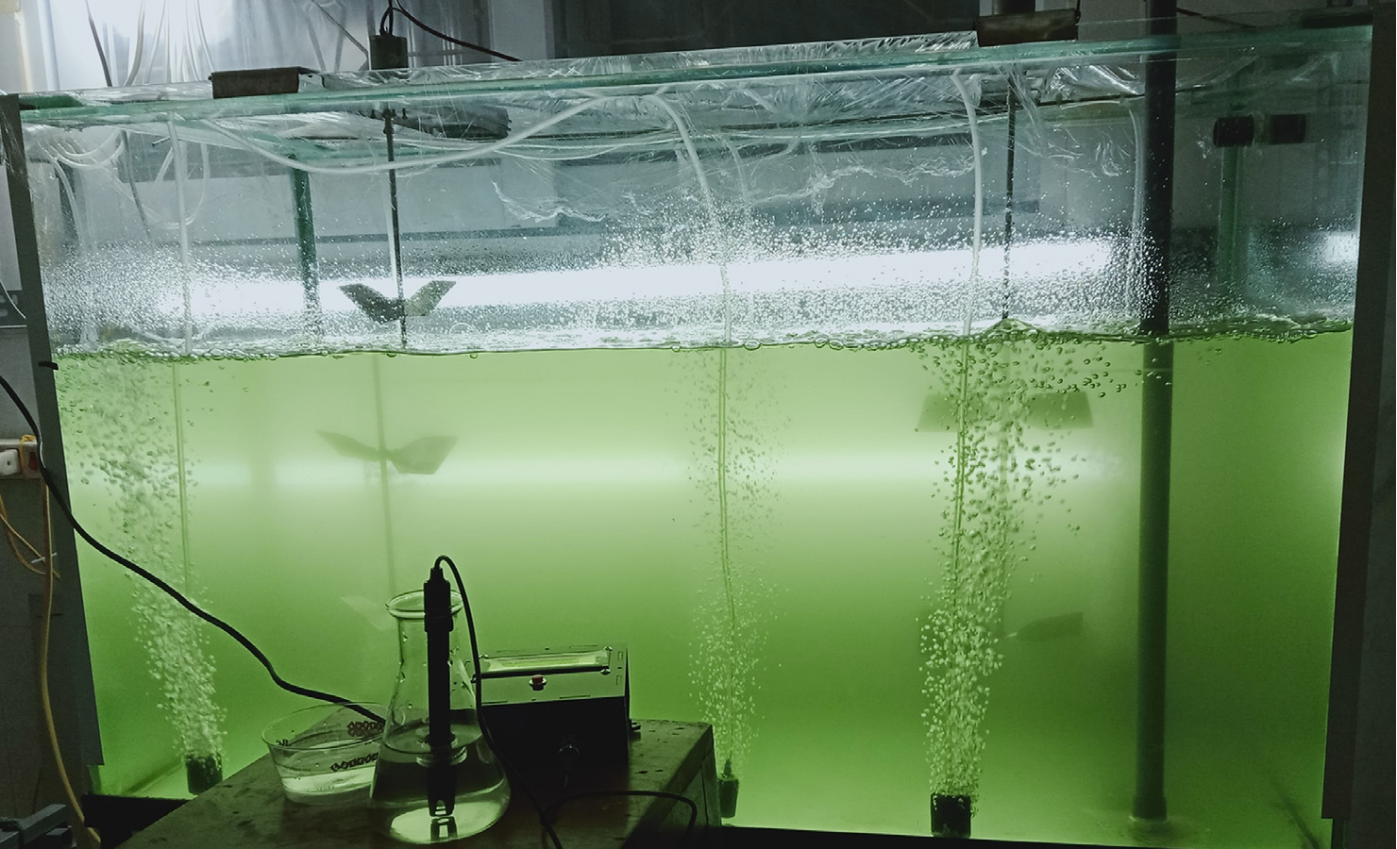
The setup for validating the monitoring system.

**Fig. 24:**
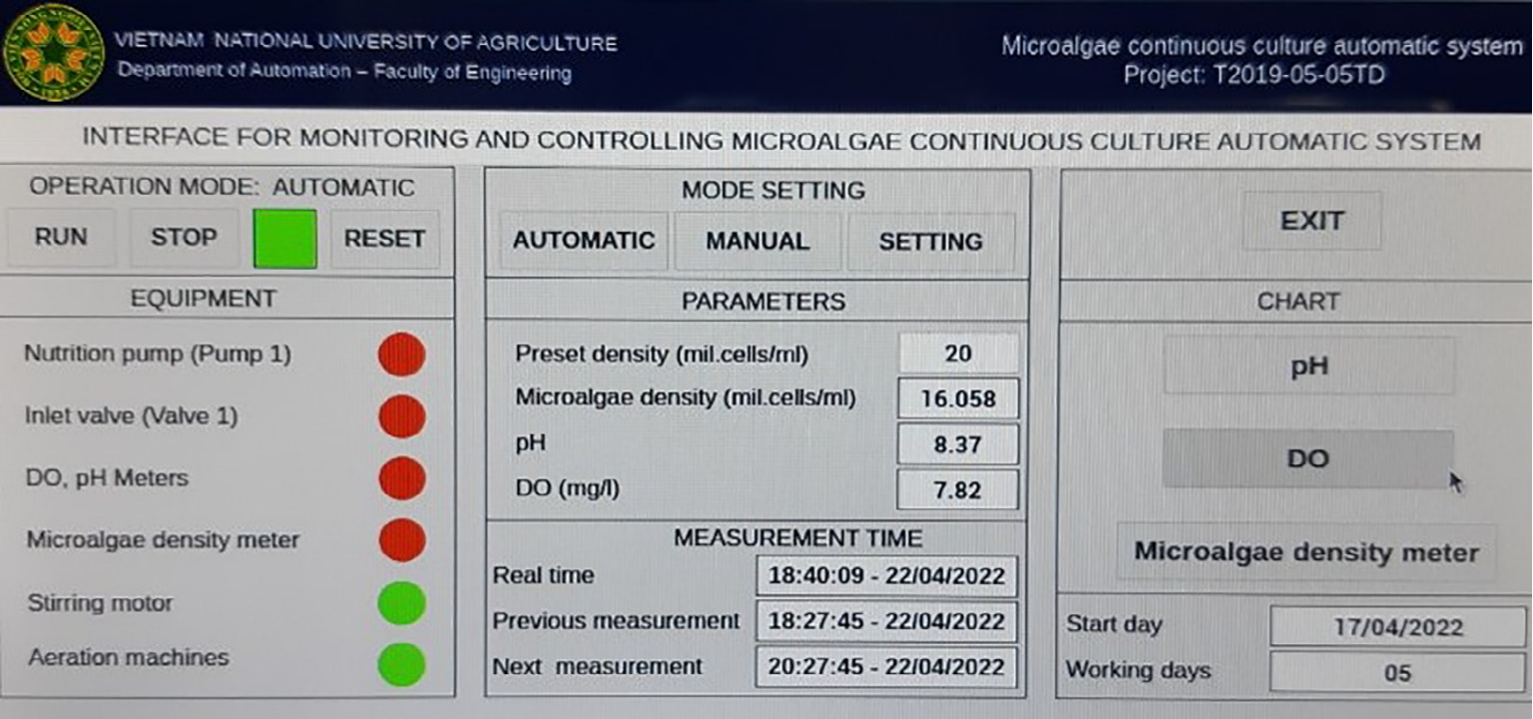
The HMI interface of the monitoring system.

In order to validate and characterize the proposed monitoring system, it was implemented in a continuous culture model, where the culture tank has dimensions of 600 mm × 450 mm × 1000 mm. In the culture tank, the microalgal strain named *Chlorella vulgaris* was cultivated. The setup of the continuous model for growth the microalgae is demonstrated in Fig. 23. When the monitoring system was run, the HMI interface for displaying the measurements was captured and the snapshot is illustrated in Fig. 24. It is noted that the HMI was also designed to operate and control some other systems in the continuous model such as ventilation, stirring, …which are beyond scope of this paper. Some results of the pH, DO and microalgal density measurements gathered by the designed monitoring system are summarized as follows. Table 1.

**Table 1:** Design files.

**Design filename**	**File type**	**Open source license**	
Dark box	PDF, CAD	CC-By Attribution 4.0 International	
Microalgae density circuit	PDF	CC-By Attribution 4.0 International	
Sample container 2	PDF, CAD	CC-By Attribution 4.0 International	
pH and DO measurement circuit	PDF	CC-By Attribution 4.0 International	
Central control unit	INO	CC-By Attribution 4.0 International	
pH DO measurement	INO	CC-By Attribution 4.0 International	
Automatic mode interface	UI	CC-By Attribution 4.0 International	
Microalgae graph interface	UI	CC-By Attribution 4.0 International	
pH graph interface	UI	CC-By Attribution 4.0 International	
DO graph interface	UI	CC-By Attribution 4.0 International	
Main	PY	CC-By Attribution 4.0 International	

### 7.1 pH sensor validation

To verify the pH sensor, the experiments of deploying the monitoring system were conducted in two different solutions with known pH values of 4.01 and 7.00, respectively. For each solution, 10 measurements were collected and the results are summarized in Table 2. As compared with the known pH values, the errors of the measurements obtained by the proposed monitoring system are 0.012 (0.300%) and 0.014 (0.200%), respectively.

**Table 2:** pH sensor validations in the solutions with known pH values.

**No.**	**Solution with pH** **=** **4.01**			**Solution with pH** **=** **7.00**					
	**pH**	▵**pH**	δ**pH (%)**	**pH**	▵**pH**	δ**pH (%)**			
1	4.02	0.01	0.25	6.98	0.02	0.29			
2	3.99	0.02	0.50	7.00	0.00	0.00			
3	4.01	0.00	0.00	6.99	0.01	0.14			
4	4.00	0.01	0.25	6.98	0.02	0.29			
5	4.02	0.01	0.25	6.98	0.02	0.29			
6	3.98	0.03	0.75	7.01	0.01	0.14			
7	4.00	0.01	0.25	6.98	0.02	0.29			
8	3.99	0.02	0.50	6.99	0.01	0.14			
9	4.00	0.01	0.25	7.01	0.01	0.14			
10	4.01	0.00	0.00	6.98	0.02	0.29			

### 7.2 DO sensor validation

Similar to the pH sensor, the DO sensor in the proposed device was also evaluated in the different solutions. In each solution, while a measurement was taken by the developed equipment, another measurement obtained by HI8043 meter was also recorded for the comparison purpose. The measured results are tabulated in Table 3, where the errors between the results correspondingly obtained by the monitoring system and HI8043 meter are calculated accordingly. The mean error in eight measurements is 0.303 (3.800%).

**Table 3:** DO sensor validations in the different solutions where the resulted measurements are compared with those obtained by HI8043 meter.

**Sample No.**	**HI8043 meter (mg/l)**	**The developed system (mg/l)**	▵**DO**	δ**DO (%)**
1	8.23	8.20	0.03	0.37
2	8.14	8.09	0.05	0.62
3	8.10	8.05	0.05	0.62
4	7.69	7.68	0.01	0.13
5	7.19	7.25	0.06	0.83
6	8.28	8.25	0.03	0.36
7	8.30	8.37	0.07	0.84
8	8.28	8.30	0.02	0.24

### 7.3 Microalgal density validation

**Fig. 25:**
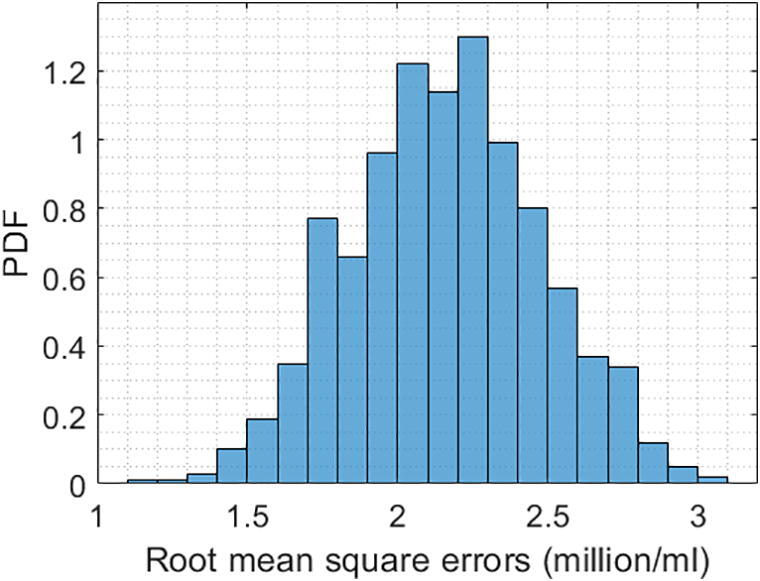
The microalgal density root mean square errors between the measurements obtained by the monitoring system and the corresponding ground truths.

The microalgal density measurement unit was verified by conducting the experiments in 25 different microalgae solutions, where their corresponding density ground truths range from 10.5 to 42.3 million cells per ml. The ground truth of the microalgae density in each solution was obtained by the manual direct microscopic method as discussed in Section 6.1.3. For each solution, the unit captured 3 images, which were then interpreted into 3 microalgal density values by the monitoring system. The root mean square errors between the microalgae density interpretations and the ground truths were computed and summarized in a histogram as depicted in Fig. 25. On average, the measurement accuracy obtained by the developed device as compared with the ground truth is about 8.6% (±1.8%).

## 8 Conclusions

The paper has provided a detailed design of a low-cost monitoring system that can be employed to observe pH, DO and microalgal density parameters in a continuous chamber of culturing microalgae. The hardware of the system has been described while its designed components have also been delineated. Moreover, instructions of how to build the device from the designed components have been presented; and the guideline on how to efficiently operate the proposed equipment has also been provided. All the design files and material bills are attached for the reproducing purpose. The monitoring system was validated in a real-life microalgae chamber where the obtained results indicate its promising practicality.

## CRediT authorship contribution statement

**Dung Kim Nguyen:** Conceptualization, Investigation, Methodology, Data curation, Software, Validation, Writing - original draft. **Huy Quang Nguyen:** Investigation, Validation, Writing - review & editing. **Huyen Thuy T. Dang:** Investigation, Validation, Writing - review & editing. **Viet Quoc Nguyen:** Software, Validation. **Linh Nguyen:** Investigation, Software, Validation, Writing - review & editing.

## Declaration of Competing Interest

The authors declare that they have no known competing financial interests or personal relationships that could have appeared to influence the work reported in this paper.

## References

[1] Lavens P., Sorgeloos P. (1996).

[2] Muller-Feuga A. (2000). The role of microalgae in aquaculture: Situation and trends. J. Appl. Phycol..

[3] Peter A.P., Chew K.W., Koyande A.K., Yuk-Heng S., Ting H.Y., Rajendran S., Munawaroh H.S.H., Yoo C.K., Show P.L. (2021). Cultivation of chlorella vulgaris on dairy waste using vision imaging for biomass growth monitoring. Bioresour. Technol..

[4] Gao F., Li C., Yang Z.-H., Zeng G.-M., Feng L.-J., Zhi Liu J., Liu M., Wen Cai H. (2016). Continuous microalgae cultivation in aquaculture wastewater by a membrane photobioreactor for biomass production and nutrients removal. Ecolog. Eng..

[5] Matteau D., Baby V., Pelletier S., Rodrigue S. (2015). A small-volume, low-cost, and versatile continuous culture device. PLOS ONE.

[6] Peter A.P., Koyande A.K., Chew K.W., Ho S.-H., Chen W.-H., Chang J.-S., Krishnamoorthy R., Banat F., Show P.L. (2022). Continuous cultivation of microalgae in photobioreactors as a source of renewable energy: Current status and future challenges. Renew. Sustain. Energy Rev..

[7] Laing I. (1991).

[8] Sananurak C., Lirdwitayaprasit T., Menasveta P. (2009). Development of a closed-recirculating, continuous culture system for microalga (tetraselmis sueeica) and rotifer (brachionus plicatilis) production. ScienceAsia.

[9] Naumann T., Çebi Z., Podola B., Melkonian M. (2012). Growing microalgae as aquaculture feeds on twin-layers: A novel solid-state photobioreactor. J. Appl. Phycol..

[10] Metsoviti M., Papapolymerou G., Karapanagiotidis I., Katsoulas N. (2019). Comparison of growth rate and nutrient content of five microalgae species cultivated in greenhouses. Plants.

[11] Tham P.E., Ng Y.J., Vadivelu N., Lim H.R., Khoo K.S., Chew K.W., Show P.L. (2022). Sustainable smart photobioreactor for continuous cultivation of microalgae embedded with internet of things. Bioresour. Technol..

[12] Lim H.R., Khoo K.S., Chia W.Y., Chew K.W., Ho S.-H., Show P.L. (2022). Smart microalgae farming with internet-of-things for sustainable agriculture. Biotechnol. Adv..

[13] V.A. Thiviyanathan, H.R. Lim, P.E. Tham, P.L. Show, Microalgae for Environmental Biotechnology, CRC Press, 2022, Ch. How Far Has the Development for Industrial Internet of Things (IoT) in Microalgae? p. 30.

[14] S. Ali, K.S. Khoo, H.R. Lim, H.S. Ng, P.L. Show, Microalgae for Environmental Biotechnology, CRC Press, 2022, Ch. Smart Factory of Microalgae in Environmental Biotechnology, p. 30.

[15] Metsoviti M., Papapolymerou G., Karapanagiotidis I., Katsoulas N. (2019). Effect of light intensity and quality on growth rate and composition of chlorella vulgaris. Plants.

[16] Blair M.F., Kokabian B., Gude V.G. (2014). Light and growth medium effect on chlorella vulgaris biomass production. J. Environ. Chem. Eng..

[17] Fancher N. (2018). Chroma: A Photographer’s Guide to Lighting with Color. Rocky Nook.

[18] Nguyen D.K., Nguyen L., Viet Le D. (2021). A low-cost efficient system for monitoring microalgae density using gaussian process. IEEE Trans. Instrum. Meas..

[19] Nguyen L., Nguyen D.K., Nghiem T.X., Nguyen T. (2022). Least square and gaussian process for image based microalgal density estimation. Comput. Electron. Agricul..

[20] Goldman J.C., Azov Y., Riley C.B., Dennett M.R. (1982). The effect of ph in intensive microalgal cultures. i. biomass regulation. J. Exp. Mar. Biol. Ecol..

[21] A. Cortés Téllez, S. Sánchez-Fortún, M. García-Pérez, B.-C. Mc, Effects of ph on the growth rate exhibited of the wild-type and cd-resistant dictyosphaerium chlorelloides strains, Limnetica 37. doi:10.23818/limn.37.19.

[22] Kazbar A., Cogne G., Urbain B., Marec H., Le-Gouic B., Tallec J., Takache H., Ismail A., Pruvost J. (2019). Effect of dissolved oxygen concentration on microalgal culture in photobioreactors. Algal Res..

